# Ancient DNA insights into pathogens and their hosts

**DOI:** 10.1038/s41576-025-00912-4

**Published:** 2025-12-03

**Authors:** Kelly E. Blevins, María C. Ávila-Arcos, Verena J. Schuenemann, Anne C. Stone

**Affiliations:** 1 Department of Archaeology, University of Durham, Durham, UK; 2 Center for Bioarchaeological Research, Arizona State University, Tempe, AZ, USA; 3 Laboratorio Internacional de Investigación sobre el Genoma Humano, Universidad Nacional Autónoma de México, Querétaro, Mexico; 4 Department of Environmental Sciences, University of Basel, Basel, Switzerland; 5 Institute of Evolutionary Medicine, University of Zurich, Zurich, Switzerland; 6 Department of Evolutionary Anthropology, University of Vienna, Vienna, Austria; 7 Human Evolution and Archaeological Sciences (HEAS), University of Vienna, Vienna, Austria; 8 School of Human Evolution and Social Change, Arizona State University, Tempe, AZ, USA; 9 Center for Evolution and Medicine, Arizona State University, Tempe, AZ, USA; 10 Institute of Human Origins, Arizona State University, Tempe, AZ, USA

## Abstract

Ancient DNA research of pathogens and their animal and human hosts provides unique insights into past health, the changes that led to pathogen jumps and pandemic outbreaks, the timing of such events, and the factors (both genetic and cultural) that affect pathogen success and human survival and recovery. Technological improvements and the increasing number of ancient samples analyzed have enabled an unprecedented investigation into pathogen evolution. Pathogen emergence and adaptation are constant, but the mechanisms underlying pathogen success as well as host susceptibility and resistance are often only observable in time series data. When contextualized with historical and archaeological data, ancient genomes provide a novel approach for detecting how sociocultural and biological changes interact. In this review, we examine this recent research as well as the challenges that remain. We focus on the zoonoses that became human endemic or intermittent plagues, the relationship of these to human mobility and migration (particularly during the Colonial Period), the signals of selection and population change that are found in both the hosts and the pathogens, and the potential for archival samples to reveal more about these histories.

## Introduction

Ancient DNA research of pathogens and their hosts reveals the evolutionary history and pressures of the greatest selective agents affecting us. Such analyses also show why the study of the past is relevant today: the process of pathogen emergence and adaptation is constant and requires data collected over time and space for full understanding. The growing application of One Health approaches in the medical, biological and social sciences have fostered a recent trend in ancient pathogen research to search for pathogens in animal hosts. The One Health framework recognises human health and well-being as innately linked to that of their ecosystem, including the health of animals and plants. This pivot from human-centric ancient pathogen genomics has substantially broadened our perspective on the contexts of zoonotic disease transmission, drivers of host adaptation, and role of human behaviour and mobility on pathogen evolution.

**Figure 1:**
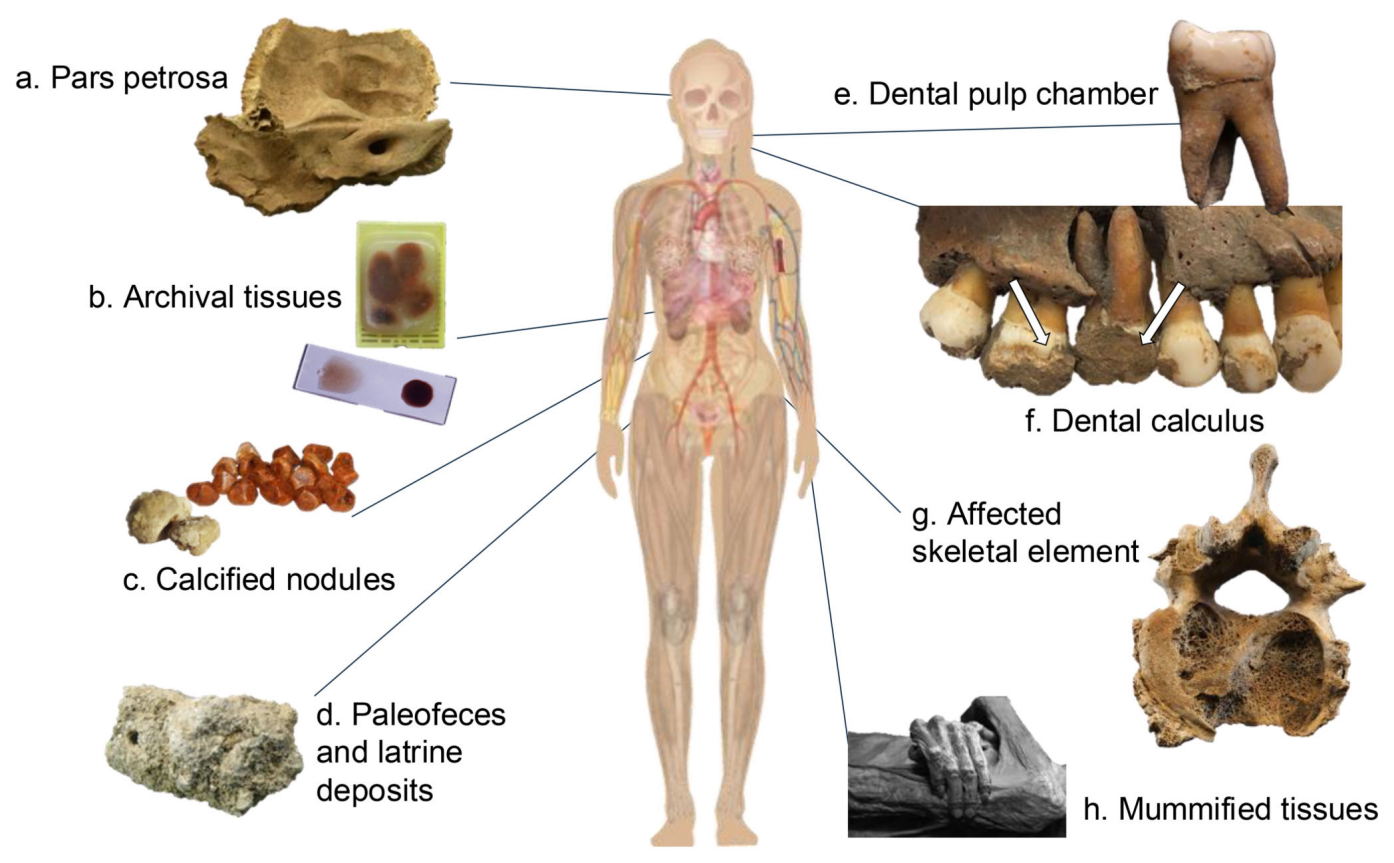
Strategies for recovering pathogen DNA from archival and archaeological substrates a. This element is prioritized for host DNA due to the dense cortical structure of the inner ear that preserves DNA well. Pathogens are unlikely to be present here at the time of death, but hepatitis B genomes have been recovered from large opportunistic screening efforts. Other isolated cases of pathogen recovery from the pars petrosa include *Treponema pallidum* from a human infant and *Brucella melitensis* from an adult sheep ^36,52,74,75^. b. Archival and museum specimens are being increasingly targeted as unique sources of pathogen genomic material, particularly for RNA viruses that do not preserve in archaeological substrates. Preserved lung and intestinal tissues, formalin-fixed paraffin embedded tissue biopsies, and even microscopy slides have yielded whole pathogen genomes. ^64,66,76–78^. c. Calcified nodules from historical and archaeological remains are promising substrates for recovering localized pathogens. Kidney stones and calcified pelvic and lymph nodes are the only source of ancient *B. melitensis* genomes from humans ^25,32,34,35^. d. Paleofeces are a rich source of information for host microbiota. Recently, mitochondrial genomes from the gastrointestinal parasites *Ascaris lumbricoides, Trichuris trichuria, T. muris*, and *Dicrocoelium dendriticum* have been recovered from latrine deposits^79,80^. e. Teeth are also a common sampled substrate for host DNA, and some of the first ancient pathogen genomes were isolated from the dental pulp chamber, such as *Yersinia pestis*. It is thought that pathogens causing sepsis or localized infections in the oral cavity and blood borne pathogens can be detected in this substrate. *Salmonella enterica, Haemophilus influenzae, Borrelia recurrentis, Mycobacterium leprae*, hepatitis B virus, human parvovirus 19, and herpes simplex virus have been isolated from tooth samples ^8,14,37,38,52,61,75,81–89^. f. Calculus is mineralized dental plaque that is commonly observed on archaeological remains. Calculus has been a source of opportunistic pathogens of the oral microbiome, such as *Tannerella forsythia*, and pathogens localized to the orofacial region, such as *M. leprae*, and pathogens that cause chronic respiratory disease, such as *Klebsiella pneumoniae* ^54,55,90,91^. g. Skeletal lesions can act as a signpost that the pathogens was present at the time of death. Paleopathology-informed sampling has also been the most successful for recovering ancient *Treponema pallidum* and *Mycobacterium tuberculosis* complex DNA. Indeed, all ancient *M. tuberculosis* genomes from skeletal material have been recovered from vertebral bodies or ribs with pathological lesions ^24,44,47,74,92,93^. h. Mummified tissues offer a unique opportunity to recover pathogens isolated to the soft tissues that normally do not preserve in the archaeological record. A *Helicobacter pylori* genome was recovered from a mummified gastrointestinal tract and variola virus from mummified skin^94–96^.

Additionally, the methodological improvements in ancient DNA analyses (i.e. see ^1^) and growing diversity of sampled substrates (Fig 1.) have resulted in publication of hundreds of ancient pathogen genomes as well as host data sufficient for the assessment of susceptibility and resistance of humans. Critically, ancient genomes are complemented by historical and archaeological data that characterize the social and cultural responses to disease, key components affecting pathogen spread or eradication. Further, archival medical collections, while more recent than many samples from archaeological contexts, can provide access to historical samples that are often accompanied by important documentation about symptoms and patient histories in the era prior to modern medical treatments.

**Figure 2:**
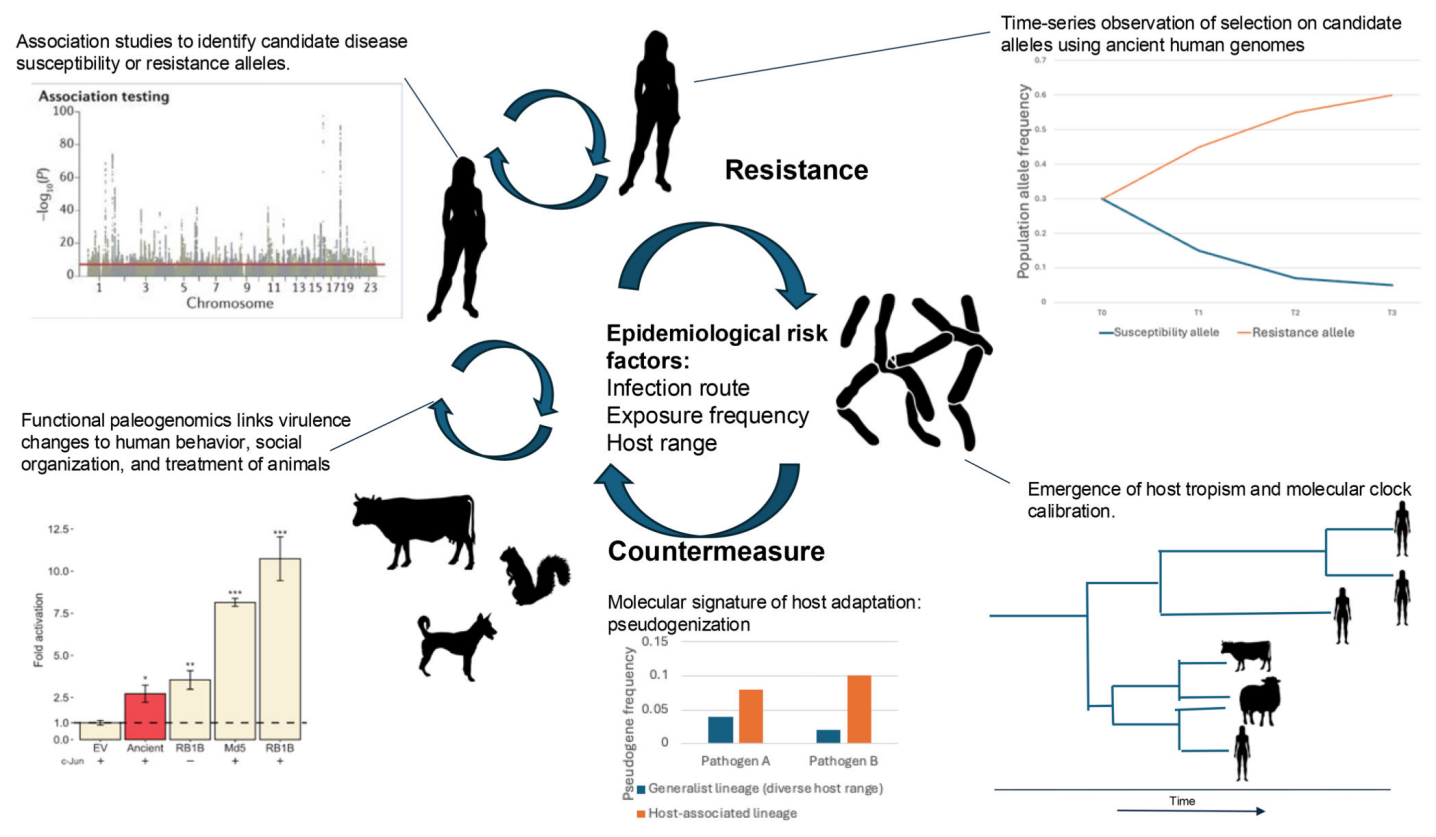
Host-pathogen interactions observable through time series data (ancient genomics)

The increasing amount of modern and ancient genome data from pathogens reveals how they adapt to new hosts and evade immune systems ^3^. Zoonotic jumps are the means whereby new pathogens are introduced, but subsequent adaptation to the new host is typically required for persistence and endemicity. Adaptation to a new host occurs through a cycle of host resistance and pathogen countermeasure that is mediated by epidemiological factors such as infection route, exposure frequency, and host range (Fig. 2). A microbe’s trajectory from opportunistic to obligate pathogen can include genome reduction or pseudogenization, likely because biomolecular secretion is energetically costly and likely to alert the host’s immune system. Emergence of host tropism can also be observed using phylogenomics and molecular clock estimation calibrated by the dates of archaeological or historical samples. To understand the effects of changes, functional paleogenomics can be used to synthesize ancient genes and compare their expression to modern analogs ^4,5^.

**Figure 3:**
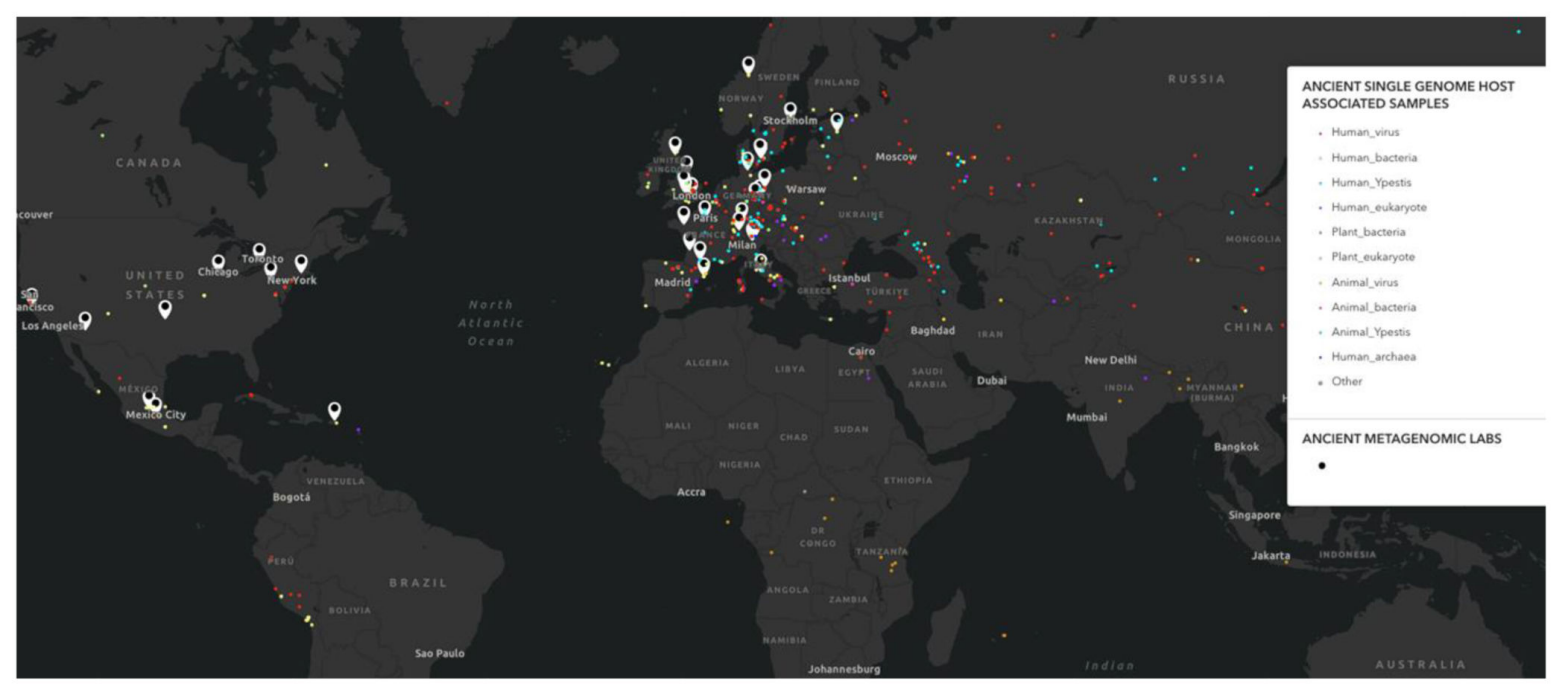
Interactive map depicting locations of ancient genomes recovered from pathogens discussed in this review. Metadata were gathered from AncientMetaGenomeDir ^97^. See https://unigisal.maps.arcgis.com/apps/instant/basic/index.html?appid=59a915f0c9ce432d97053affcfb62fdb

While most ancient pathogen genomes are from *Yersinia pestis*, the causative agent of plague and responsible for hundreds of millions of deaths (reviewed in ^2^), recent studies on ancient, viral, bacterial, and eukaryotic pathogens have provided a more diverse appreciation of the molecular mechanisms of pathogenicity and the connection between human cultural practices and pathogen diversification (Fig. 3, see web-based interactive map). In this Review, we integrate these diverse insights with a focus on the mechanisms of pathogen adaptation and host-pathogen coevolution, the zoonoses that succeeded in becoming human endemic or intermittent plagues, the impact of human mobility and migration (particularly during the Colonial Period) on pathogen distributions, and the potential for archival samples to reveal more about recent pathogen histories prior to the widespread use of antibiotics and vaccines.

## Mechanisms of pathogen adaptation and host-pathogen coevolution

The intensive bidirectional selective pressures of hosts and pathogens (i.e. host-pathogen interactions or coevolution) leave signals in their genomes. Time series ancient DNA data provide a novel means of examining these interactions, because subsequent demographic and selective events can obscure adaptive signals in modern genomes. These dynamics over the long and short term can be examined to assess how genetic changes enable some pathogens to establish in a new host and become endemic and assess which host variants result in greater resistance to disease. Recent work has yielded such insights into human infections, infections of domestic plants and animals, as well as the evolution of host resistance.

### Human infections

Genomic analyses of Variola virus (VARV), the causative agent of smallpox, and *Y. pestis* illustrate how these changes occur in viruses and bacteria over time as a result of selective pressures in new hosts (e.g. ^6,7^). For VARV, high coverage genomes recovered from European burials dating from 600-1000 CE show changes in gene content over the course of the last few millennia ^8^. Exactly how these changes facilitate human infection and virulence is not well-understood, but the genome data from VARV as well as its closest relatives (camelpox and taterapox) point to a common ancestor with a larger genome. Subsequent gene loss appears to be linked to virulence and host specificity ^8,9^.

The changes in the *Y. pestis* genome are the earliest and best documented by ancient DNA. Surprisingly, genomes dating to the Late Neolithic and Bronze Age, prior to the three historical plague pandemics, show that *Y. pestis* circulated from central Asia to Europe millennia earlier than previously thought ^10–14^. These genomes also reveal that the murine toxin gene (*ymt*), which enhances the survival of the bacteria in the flea gut and expands host range ^15^, was acquired fairly late in the Bronze Age. The early strains also show a patchwork of variation that ultimately results in the full set of changes that facilitate flea-to-human transmission. This suggests that in early outbreaks, the flea route of transmission would not have been effective, and the plague in humans may have been transmitted via food, inhalation, or fecal-orally.

### Infections of domesticated plants and animals

Pathogens affecting domesticated plants and animals also show signals of selection ^4,16–18^, sometimes linked to outbreaks resulting in significant economic losses and famine in human populations. The most famous of these is the potato blight, caused by *Phytophthora infestans*. Analyses of the strain causing the Irish potato famine (HERB-1) revealed that its set of effector genes caused so much damage because of a lack of corresponding plant immune receptors that would detect them in the lumper potato (the primary cultivar) ^17^. Subsequent diversification of *P. infestans* lineages show increased polyploidy (rather than specific genes with clear signals of selection) as important for enhanced variation in the effector genes of this eukaryotic pathogen.

Ancient pathogen genomes have also highlighted how recent human behavior and pathogen adaptation has impacted animal health. One study investigated Marek’s disease virus (MDV), a herpes virus that today causes tumor formation and high mortality in chickens, from almost 1000 Eurasian chicken bones and found 15 that were positive for the viral DNA^4^. Analyses of the seven ancient genomes with sufficient coverage, ranging in age from the 10th to 19th centuries, showed that they were basal to modern strains and demonstrated that gene loss or gain was not responsible for the two independent increases (in Eurasia and the Americas) in MDV virulence during the 20th century. Instead, positive selection was detected in almost 50 gene regions of which several have been associated with virulence today. These include the Meq oncogene, essential for tumor formation. Using a functional paleogenomics approach, the authors synthesized a version of this gene found in a strain dating to ~1800 CE and compared its protein expression to that of modern MDV isolates. Transactivation by the ancient sequence was 67-75% lower than that of the modern MDV strains, indicating that it was unlikely to stimulate tumor formation in affected chickens. Though the virus has been circulating in Europe for ~1,000 years, evolutionary dating analysis links this increase in virulence to the onset of factory farming in the early 1900s.

### Host resistance

In hosts, candidate genes that confer susceptibility or resistance can be detected through association studies in modern populations for which host genomic and disease outcome data exist. Using ancient DNA, the allele frequency change in these candidate genes can be tracked through time and the selective pressure of a pathogen can be inferred. Many natural selection signatures in the human genome are linked to pathogen-driven pressures ^19–22^. Selective pressures are easier to identify at loci with larger effects and in well sampled regions of the world. For example, TB was an endemic pathogen in Europe for thousands of years, as demonstrated by osteological and historical data (e.g, ^23^). Evolutionary dating of the pathogen complex suggests it originated in the late Neolithic or Bronze age, but the potentially more virulent L4 strains may have appeared in Europe ~ 900 - 2500 years ago with origins in Africa or Europe ^24,25^. These dates are supported by signals of selection in ancient European populations. Specifically, Kerner and colleagues^26^ found that the frequency of a European allele of the TYK2 gene that causes homozygotes to have higher risk of developing clinical TB began to drop dramatically after the Bronze age.

Differentiating signatures of positive selection from genetic drift in genomic data remains challenging ^27^. The complex demographic histories of human populations combined with small study sample sizes and inherent enrichment biases in ancient DNA data acquisition can manifest as false positive results. For example, the high mortality during the Black Death suggests that signatures of natural selection should be apparent in the genomes of people who survived the pandemic ^28,29^. Klunk and colleagues^29^ examined immune loci in people who lived before, during and after the pandemic in London (n= 318) and Denmark (n = 198). They found four regions that appeared to be strongly linked to differential survival and generated functional data from *in vitro* infection experiments. The strongest response was seen in a variant of the ERAP2 gene (involved in antigen presentation) with fivefold increased expression. After randomization testing and correcting for genotype likelihoods biased by uneven coverage typical of ancient DNA, Barton et al. ^30^ found no evidence for allele frequency change in the ERAP2 gene before and after the Black Death. The decline of the Black Death could be related to changes in both host and pathogen (as well as cultural changes). Ancient genome analyses also offer further insight into why the Black Death petered out in the 18th century CE. Specifically, ^31^ found that genomes in later strains postdating the pandemic still had two pPCP1 plasmids, but only one of these contained the *pla* gene, which plays an important role in virulence.

### Zoonoses and zoonotic potential

Although zoonotic jumps are the primary way new pathogens are introduced to human populations, few animal-adapted pathogens have been as extensively studied as those with humans as the primary host. This bias is also reflected in ancient pathogen genomics, but a recent shift in focus to searching for pathogens in non-human hosts has revealed how human behavior has shaped pathogen evolution in animals and highlighted the permeability of human-wildlife interface for pathogen transmission.

### Animal domestication and husbandry as drivers of pathogen evolution

The establishment of large permanent settlements and animal domestication during the Neolithic provided a novel adaptive landscape for opportunistic and generalist pathogens and increased the risk of emerging infectious diseases. Indeed, ancient pathogen genomes from humans and their domesticates from archaeological contexts have revealed that human-animal interactions precipitated host specialization and increased virulence.

Because of its significant health and economic impact and ability to leave characteristic skeletal changes in infected humans, *Brucella* was an early target of ancient pathogen DNA studies ^32^. *Brucella* is a genus of intracellular pathogens that cause brucellosis. There are 12 recognized species that infect mammals, but *B. abortus* (primary host cattle) and *B. melitensis* (primary host sheep and goats) have the largest impact on animal health and the most zoonotic potential to infect humans today ^33^. *Brucella* causes abortion and infertility in infected animals and is readily transmitted to humans and other animals through contact with infected milk and aborted fetuses or placenta. Only two ancient *B. melitensis* genomes have been recovered from archaeological human remains, likely due to a low bacterial load in infected human skeletal tissues ^32,34,35^.

Despite difficulties recovering *Brucella* from human remains, one study^36^ recovered a *B. melitensis* genome from a Neolithic Anatolian sheep cranial bone (6057-5913 cal BCE).

Evolutionary dating of the phylogeny calibrated by this genome indicates that *B. melitensis* and *B. abortus* diverged between 8,246-7,497 BCE, corresponding to the intensification of pastoralism across Neolithic Southwest Asia. Phylogenetically, the Neolithic *B. melitensis* genome falls soon after the divergence of *B. melitensis* and *B. abortus* and contains three pseudogenization events absent in *B. abortus*, highlighting the early development of host tropism in this lineage.

*Salmonella enterica* causes gastroenteritis and enteric fever in infected individuals. Key and colleagues^37^ demonstrated that Neolithic foragers contracted *S. enterica* lineages that have broad host ranges, whereas Neolithic agropastoralists had more closely genetically related lineages with high pseudogenization. Further, the ancient *S. enterica* Paratyphi C genetic diversity from Neolithic agropastoralists includes modern pig lineages. This suggests that the pathogen evolved to both hosts concurrently or that *S. enterica* Paratyphi C passed from humans to their domesticated pigs.

*Yersinia pestis* was widespread in Late Neolithic and Bronze Age northern European humans despite lack of genetic evidence for flea-borne transmission and known rodent reservoirs ^10,12,13^. It has been hypothesized that an unknown reservoir was responsible for sporadic zoonotic spillovers or that these earlier forms of *Y. pestis* were human-human transmissible. Recent *Y. pestis* genomes from humans and a dog suggest these transmission routes were not mutually exclusive. Susat and colleagues^38^ recovered two late Neolithic *Y. pestis* genomes from modern Germany that occupy a basal position in the phylogeny and lack the pseudogenization that characterizes the later Late Neolithic and Bronze Age clade. This implies these early lineages had a wide host range. Indeed, a partial *Y. pestis* genome isolated from a dog mandible (2,950-2,500 BCE) in present-day Sweden suggests that domesticated dogs may have served as reservoirs of these generalist lineages circulating in wildlife and spread the infections to humans. Further, Seerholm et al.^39^ detected Late Neolithic *Y. pestis* in 17% of individuals buried in Neolithic tombs in modern Sweden. Since the threshold of detection is high for ancient pathogens, this high prevalence suggests plague was human-human transmissible in this region.

### The wildlife-human interface

Ancient genomics has also identified pathogen exchange across the human-wildlife interface. Tuberculosis (TB) in modern humans is caused by human-associated lineages of the *Mycobacterium tuberculosis* complex (MTBC), which likely evolved in Africa after the peopling of the Americas, precluding its introduction through initial human migrations ^24,40^. Despite this, skeletal lesions indicative of TB have been identified on Indigenous skeletons throughout the Americas ^41^. Ancient MTBC genomes from pre-contact humans show that they were infected with *M. pinnipedii*, a lineage that is isolated today only from wild and captive pinnipeds, their neighbours in captivity, and the occasional zookeeper ^42,43^. *M. pinnipedii* genomes have been recovered in human skeletons near the Peruvian coast where past populations were known to exploit seals and sea lions ^24^, as well as from skeletons ~200 km inland in modern day Colombia, suggesting that the pathogen was spread through human-to-human transmission ^44^.

*Mycobacterium leprae*, a causative agent of leprosy, is one of the few pathogens that has been isolated from archaeological human and non-human samples. Leprosy was common in medieval Europe, and hundreds of social institutions (leprosaria) for the care and treatment of the affected were established. *M. leprae* has been isolated from medieval human skeletal remains across Europe as well as modern red squirrels in Britain ^45–47^. Recently, Urban et al. ^48^ recovered *M. leprae* genomes from medieval human skeletons interred in a leprosarium and squirrel remains from the same city, which was known to have traded in squirrel fur; all genomes fall into the same lineage. Although the directionality of transmission for the studied cases is unclear, British red squirrels likely contracted *M. leprae* from humans once the pathogen was introduced into the British Isles and it may have spilled back into humans as they were reared as pets and for fur.

## The impact of human mobility and colonisation on pathogen distribution

Phylogenetic analyses of present-day pathogenic genomes reveal that some pathogens have co-evolved with humans for millennia ^49,50^. The study of these evolutionary relationships offers insights into the migration patterns of humans and the infectious diseases they have faced. While these modern-day genomic studies provide relevant knowledge, the inclusion of ancient pathogen genomes has painted a more comprehensive spatiotemporal picture of these enduring relationships and expanded our understanding of how human migrations have contributed to the distribution of pathogens and disease.

### Hepatitis B (HBV)

One example of a pathogen with extensive spatiotemporal sampling is Hepatitis B Virus (HBV). Hundreds of ancient HBV genomes have been reconstructed from human remains spanning a wide range of dates and geographical locations ^51^. The oldest HBV genomes reported so far were recovered from hunter-gatherers in America and Europe, dating to the early Holocene (~10,000 BCE) ^52^. The estimated time to the most recent common ancestor (tMRCA), calculated using both ancient ancient and modern HBV genomes, is approximately 14,000–22,000 years ago (ya). This suggests that the virus was most likely introduced to the Americas by the first humans who migrated into the continent. In Eurasia, HBV lineages spread with farming during the Neolithic, from approximately 4,000 to 2,000 BCE, followed by a turnover of strains during the Bronze Age when pastoralist steppe populations spread into Europe ^51,52^.

### Tannerella forsythia

Another microbe with a longstanding relationship with humans is the oral bacterium *Tannerella forsythia*, which is involved in periodontal disease. Ancient DNA from this pathogen has been identified in several ancient individuals, including medieval Europeans ^53^ and pre-contact Native American ancestors from Mexico and the United States ^54,55^. Since the transmission of *T. forsythia* requires close physical contact between humans, the most parsimonious explanation is that this bacterium was already established in the human populations that peopled the Americas. Interestingly, the phylogeny of *T. forsythia* reveals two distinct lineages: one formed by pre-contact genomes and another consisting of ancient and modern genomes from Europe and the United States, as well as post-contact genomes from the Americas ^54–56^. This suggests a replacement of Indigenous strains by European-introduced ones following European colonization ^54,55^.

### Smallpox and measles

*T. forsythia* is not the only pathogen for which there is evidence of introduction after European colonizers invaded territories between the 15th and 18th centuries. Many of these colonized regions, particularly in the Americas and the Pacific Islands, suffered from devastating epidemics that decimated the local populations ^57^. For example, documentary evidence from the colonial period in New Spain highlights massive epidemic outbreaks associated with smallpox, caused by VARV, and measles, caused by measles morbillivirus (MeV), as well as two outbreaks responsible for an estimated 5 to 10 million deaths termed *Cocoliztli* (‘pest’ in Nahuatl) during the 16th century ^58^. No VARV genome associated with an epidemic outbreak has been recovered in the Americas, while the recovery of ancient MeV also seems implausible given it is an RNA virus. Regarding *Cocoliztli*, there is inconclusive evidence of the pathogen(s) that could have caused it. ^59^ proposed that an endemic strain of *S. enterica* Paratyphi C caused the *Cocoliztli* outbreak at the time of contact. The disease was described as an hemorrhagic fever with death occurring within 3 to 4 days and symptoms including intense bleeding, dark urine, dysentery, acute abdominal and thoracic pain and neurological disorders, among others ^58^. Although some of the symptoms correspond to those caused by *Salmonella* infection, the discovery of this pathogen in other non-epidemic Colonial contexts (Bravo-Lopez et al., in prep), and the acuteness of the symptoms raises questions as to whether this could have been caused by an introduced hemorrhagic virus ^58^.

### Tuberculosis in the Americas

Although today TB in humans is caused predominantly by human-adapted lineages, in pre-colonial times *M. pinnipedii* was the causative agent of TB in the Americas ^24,44^. Human-to-human transmission of *M. pinnipedii* is no longer observed in present-day Americas or elsewhere, and L4 is the predominant TB lineage in the Americas today^49,60^. This suggests that there was a complete replacement of *M. pinnipedii* by L4 in the Americas. There was probably a dual mechanism for strain replacement: Indigenous people were more susceptible to strains introduced by European colonizers than they were to *M. pinnipedii*, and the high morbidity and mortality that accompanied the introduction of novel pathogens and enforced social, political, and economic change preferentially affected people already immunocompromised with *M. pinnipedii* infections, limiting its subsequent transmission.

### Parvovirus and HBV in Mexico

Genome data from other pathogens recovered from colonial contexts in Mexico, as is the case of parvovirus B19 (B19V) ^61^ and HBV ^61,62^, suggest an African origin ^61,62^. This phylogenetic placement, coupled with additional evidence from host genetics and strontium isotope analysis, suggests that these viruses and their hosts had an African origin and were introduced by Europeans during the Transatlantic Slave Trade. It is plausible that the harsh and unsanitary conditions that the enslaved Africans were subjected to by the European traders during the Middle Passage facilitated the proliferation of disease-causing pathogens. These findings thus reveal human migrations for which the documental evidence of people’s origins tends to be scarce or intentionally neglected such as those associated to the Transatlantic Slave Trade.

### Malaria-causing Plasmodium

Similarly, the analysis of ancient genomes of *Plasmodium*, the malaria-causing protozoa, from a wide array of locations and temporalities, revealed that *P. falciparum* was likely introduced to the Americas from Africa through the Trans-Atlantic slave trade ^63^. In contrast, the phylogenetic location of ancient European *P. vivax* genomes compared to modern American strains and the calculation of a last common ancestor to the 15^th^ century suggest an introduction of this pathogen to the Americas during the European conquest ^64^, an observation later supported by archaeological samples ^63^.

## The potential of archived samples

Archival samples are increasingly targeted in ancient pathogen research as they provide a unique window into the past of pathogens that are usually not accessible due to limited preservation. Most important in this context are RNA-viruses, whose unstable genomes do not preserve in archaeological samples but can be retrieved from formalin-fixed specimens curated in medical collections. Düx and colleagues ^65^ were the first to use a combination of next generation sequencing methods on a pathological case from the early 20^th^ century to study an ancient RNA virus. They reconstructed a high-coverage MeV genome dating to 1914, which allowed them to estimate its origin in the Bronze Age, around the founding of the first urban centers.

Further ancient RNA viruses were explored by Patrono and colleagues ^66^ in a study that focused on the 1918-1920 influenza pandemic. This is known as the largest influenza A virus (IAV) pandemic until today with a global spread causing between 20 million to 100 million deaths ^67^. The first ancient influenza virus was characterized more than 20 years ago using the direct PCR method ^68^; however the new methods allow for higher quality and more robust contamination tests ^66^. Through the reconstruction of the first three historical influenza virus genomes from Europe, Patrono and colleagues ^66^ were able to look at the genomic diversity of the virus between the continents as well as across the different waves during the course of the pandemic. They propose a frequent intercontinental spread of the virus during the pandemic and identify changes in host adaptation between the first and the second pandemic wave. Furthermore, they underline the importance of the recovery of more genomes to obtain a better resolution on the emergence of the 1918 pandemic. Although two additional influenza genomes have been added this year ^69^, limited recovery still hinders deciphering the viral genomics behind the 1918-1920 pandemic.

Medical and museum collections further provide an unprecedented archive into the pre-treatment era of many currently re-emerging pathogens with the potential to improve our understanding of long-term adaptations to antimicrobial treatments. For example, Van Dorp and colleagues ^64^ use medical microscope slides from Spain dating to 1944 to investigate the history of *P. vivax*, which is responsible for 42% of all cases of malaria outside Africa. Although this parasite is today mainly present in tropical and subtropical regions, it was prevalent in most of Europe until the second half of the 20^th^ century. The recovered European *P. vivax* genome already shows known resistance variants to antimalarial drugs, including Chloroquine and Sulfadoxine, pointing to an earlier use of these drugs than historically documented.

Another advantage of archival samples is their often well-preserved patient record, including exact preparation date and detailed diagnoses. These details allow an effective identification of potential cases as well as for a more detailed reconstruction of an emergence of a particular pathogen out of reach of methods like 14C dating. The highly disputed introduction of *M. leprae* to the Pacific Islands for example was resolved by the analysis of formalin-fixed paraffin-embedded biopsy blocks by Blevins and colleagues in 2020. They recovered nine *M. leprae* genomes dating to 1998–2015 representing various Pacific Islands. Based on the resulting phylogenetic pattern they could date back the introduction of *M. leprae* to premodern times.

## Conclusions and perspective

The long-term view of pathogen evolutionary history is important not just for understanding the past, but also for our present and future. Ancient pathogen genomics highlights the role of zoonotic disease transmission for introducing new pathogens into human populations and for fostering repeated exchanges and outbreaks. It also underscores the role of human behaviour in determining the risk factors for spillovers of these pathogens and can be used as cautionary tales to justify policy changes that center animal health and well-being. In addition, human trade routes and mobility, whether during periods of stress such as war, famine, or colonization or during more peaceful times, have played an important role in disease spread and continuance. The pathogen population dynamics related to these events can also be examined in concert with patterns of adaptation, both in the pathogen and the host(s). These signals of selection can illuminate the evolutionary race between humans and pathogens (before and after drug and vaccine introduction). For humans, this can provide insights into disease susceptibility or resistance for people with specific immune profiles, and for pathogens, it can suggest paths for designing “evolutionarily smarter” drugs or vaccines.

Our understanding of the evolutionary and zoonotic history of pathogens is only possible due to close collaboration among disciplines^71^. For example, it was the interdisciplinary collaboration of archaeogeneticists, historians, zooarchaeologists, and paleopathologists that enabled the recovery of the first *M. leprae* ancient genome from archaeological squirrel remains. It is becoming increasingly clear that wild animals have uncharacterized pathogens^72^, some with high zoonotic potential. Other studies have required close collaboration with microbiologists and functional geneticists to examine how variants identified in ancient pathogens affect virulence.

Archival samples from the past few centuries also play a crucial role in providing data for broader geographic distributions, body site distributions and types of pathogens that otherwise are not commonly preserved at archaeological sites. Recovering DNA (or RNA) from both archival and archaeological contexts also requires preservation and documentation of these records of the past as well as consultation with relevant communities and museums. Disease often confers stigma and sampling of individuals with disease is destructive. Both require thoughtfulness on the part of researchers and curators (Box 1).

Many challenges remain for furthering these aims. Small sample sizes and uneven spatiotemporal sampling limit higher resolution epidemiological inferences, because the recovery of ancient pathogen genomes is difficult, particularly in warmer climates where pathogen and parasite diversity is at its greatest. Additionally, it is unclear how infectious pathogens invade human and non-human skeletal tissues and where to sample to maximise the chances of recovering pathogen DNA. Only recently has there been a focus on non-human paleopathology, and even so there is often a bias toward better curation and documentation of human compared with faunal remains, especially those likely to be reservoir species such as small rodents (exp. ^48,73^).

Despite these challenges and cautions, this is an exciting time for ancient pathogen genomic research. Vibrant interdisciplinary engagement between geneticists and archaeologists will enable us to refine our understanding of the relationships between the introduction of domesticates, increased population density, and new pathogens in different regions of the world, the relationships between human outbreaks and animal reservoirs over time, how changes in human mobility fostered pathogen transmission, and the extent to which we can identify factors (social and biological) that have contributed to disease susceptibility through time.

## Figures and Tables

**Figure 4:**
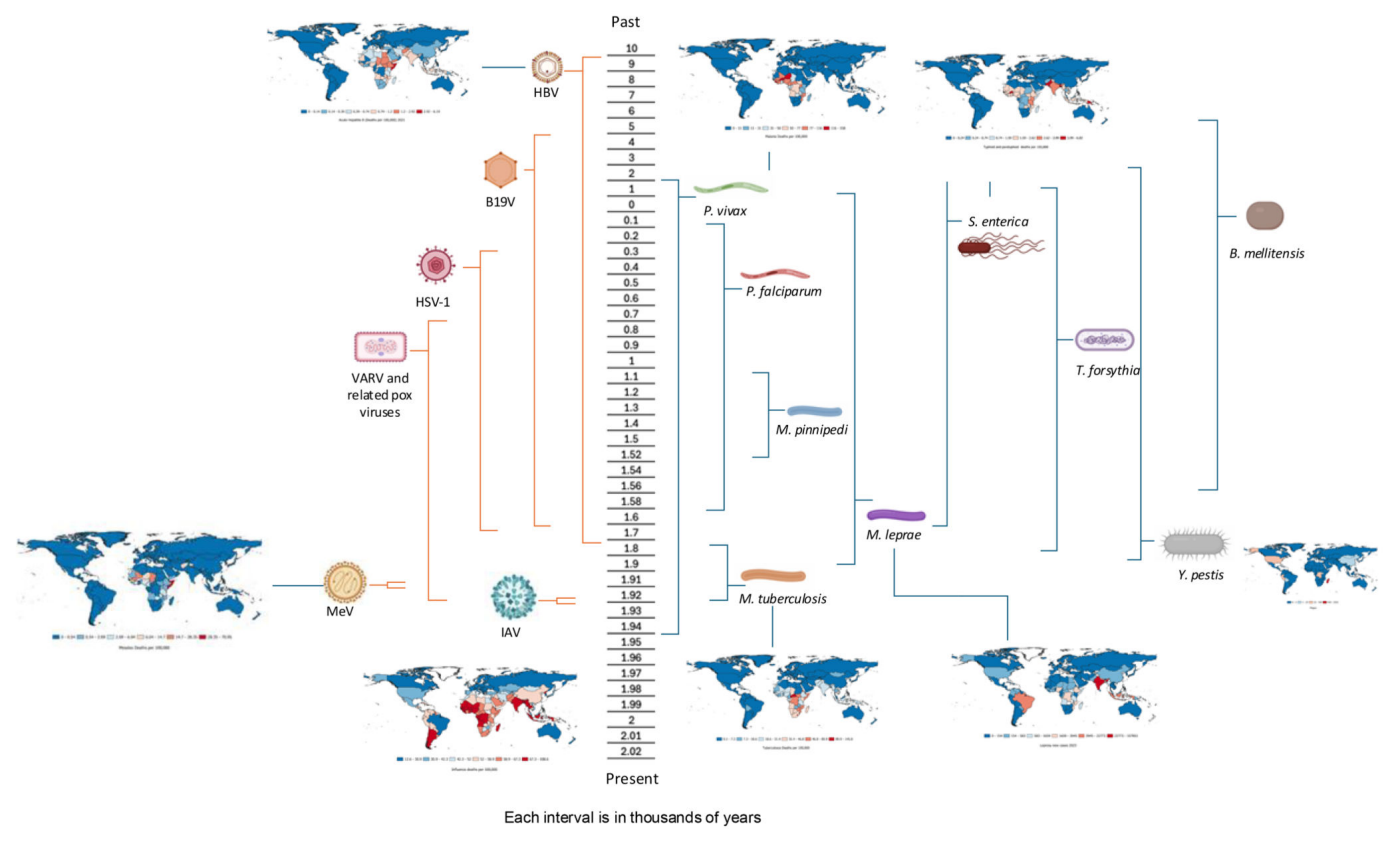
Temporal distribution of human-infecting pathogens reviewed in this study, with present-day death or incidence rates provided where data are available. The timeline, displayed in thousands of years, uses a non-linear scale to enhance clarity in visualizing the sampling ranges of each pathogen.

## Box 2 Ancient pathogen research in the context of global health

While the study of ancient pathogens shows promise for understanding the evolutionary history of disease, and it is tempting to argue that this research is fundamental to alleviating infectious diseases that threaten humans today, it is important to position ancient pathogen genomics within the context of present-day global health. The countries with the highest burden of infectious diseases suffer not from a lack of understanding of pathogen evolution, but from systemic inequities such as inadequate healthcare systems, and limited access to clean water and sanitation ^106^. For example, sub-Saharan Africa bears the highest burden of infectious diseases globally, accounting for approximately 24% of global infectious disease deaths despite having only 14% of the world’s population ^107^. This disparity is a direct legacy of colonialism, perpetuated by systemic issues that continue to disrupt local health systems and sustain economic inequalities. Moreover, the persistence of colonial practices in global health research exacerbates these inequities by prioritizing questions and methodologies that often fail to address the needs of the most affected populations ^108^.

To illustrate how power structures also influence the direction of ancient pathogen research, we can examine the disproportionate focus on *Y. pestis*. Today, however, plague has a negligible impact in Europe and a relatively minor impact in Africa compared to other infectious diseases such as HIV/AIDS, tuberculosis, and malaria, which together claim millions of lives annually in low- and middle-income countries. The overwhelming availability of well-preserved plague victims in Europe, coupled with the majority of aDNA laboratories being in Europe, has led to an excessive focus on plague at the expense of other ancient pathogens. While this focus has yielded groundbreaking methodological developments and insights into the evolutionary history and pathogenicity of *Y. pestis*, it is unlikely to have a meaningful impact on global health outcomes.

It is therefore honest to frame ancient pathogen research as a means to uncover fascinating insights into the evolution of pathogenicity, rather than as a solution to contemporary infectious disease challenges. Presenting this research as a pathway to “solve the problem of infectious diseases today” risks obscuring the systemic inequities driving infectious disease burden today.

## Glossary

One Health: A theoretical or applied approach for understanding health as an interconnection of people, animals, and the environment.
Zoonotic jump: The transmission of pathogens from animals to humans or humans to animals that causes disease in the new host.
Pseudogenization: The process by which a gene loses its function through mutations.
Biomolecular secretion: The release of molecules like proteins or toxins by cells to communicate or interact with their environment.
Host tropism: The predilection of a pathogen to infect certain hosts, tissues, or cell types.
Molecular clock: A method estimating evolutionary time based on the rate of genetic mutations.
Functional paleogenomics: The synthesis and expression of genes recovered from ancient genomes to understand the relationship between extinct genes and gene projects
Selective pressure: Environmental factors that cause a particular phenotype to be advantageous and improve reproductive success
Endemicity/endemic: Relating to a disease that is maintained in a particular population or region.
Neolithic: Broadly, the period of human history when people began living in sedentary communities and domesticating plants and animals. Different areas of the world independently developed these social, cultural, and subsistence changes.
Bronze Age: A period of human history (~3300–1200 BCE) characterized by bronze tool use, early urban civilizations, and trade networks across Europe, Asia, and Africa.
Transactivation: The process by which a protein (like a transcription factor) increases the expression of a gene.
Allele frequency change: The fluctuation of how common an allele is in a population caused by the forces of selection, drift, or gene flow.
TYK2: A gene that encodes a protein involved in immune signaling pathways and plays a role in inflammation and defense against infections.
Genetic drift: Changes in allele frequencies caused by the random chance that individuals within a population do not reproduce; genetic drift significantly impacts small populations.
Black Death: A plague pandemic (1346–1353 CE) caused by *Yersinia pestis* that killed 75–200 million people across Eurasia and North Africa.
Plasmids: Small, circular DNA molecules in bacteria that can carry genes enabling antibiotic resistance or increased virulence.
Emerging infectious diseases: An infectious disease that appears in a new host or population, often as a result of a zoonotic transmission.
Virulence: The degree of damage a microbe causes to its host.
Brucellosis: A bacterial disease causing fever, joint pain, and fatigue in humans. Humans are infected with animal-adapted species usually via a zoonotic transmission from livestock.
Evolutionary dating: A method to infer the timing of evolutionary events using genomic data and the estimation of mutation rate.
tMRCA: Time to Most Recent Common Ancestor is an estimate of when two or more lineages last shared a common ancestor.
Strontium isotope analysis: A common archaeological method to compare the ratio of strontium isotopes in skeletal tissues to that of the bedrock to infer ancient human or animal mobility and geographic origins.
